# Performance of a Full-Coverage Cervical Cancer Screening Program Using on an Artificial Intelligence– and Cloud-Based Diagnostic System: Observational Study of an Ultralarge Population

**DOI:** 10.2196/51477

**Published:** 2024-11-20

**Authors:** Lu Ji, Yifan Yao, Dandan Yu, Wen Chen, Shanshan Yin, Yun Fu, Shangfeng Tang, Lan Yao

**Affiliations:** 1 School of Medicine and Health Management Tongji Medical College of Huazhong University of Science and Technology Wuhan China; 2 The Fifth Hospital of Wuhan Wuhan China; 3 Landing Artificial Intelligence Industry Research Institute Wuhan China

**Keywords:** full coverage, cervical cancer screening, artificial intelligence, primary health institutions, accessibility, efficiency

## Abstract

**Background:**

The World Health Organization has set a global strategy to eliminate cervical cancer, emphasizing the need for cervical cancer screening coverage to reach 70%. In response, China has developed an action plan to accelerate the elimination of cervical cancer, with Hubei province implementing China’s first provincial full-coverage screening program using an artificial intelligence (AI) and cloud-based diagnostic system.

**Objective:**

This study aimed to evaluate the performance of AI technology in this full-coverage screening program. The evaluation indicators included accessibility, screening efficiency, diagnostic quality, and program cost.

**Methods:**

Characteristics of 1,704,461 individuals screened from July 2022 to January 2023 were used to analyze accessibility and AI screening efficiency. A random sample of 220 individuals was used for external diagnostic quality control. The costs of different participating screening institutions were assessed.

**Results:**

Cervical cancer screening services were extended to all administrative districts, especially in rural areas. Rural women had the highest participation rate at 67.54% (1,147,839/1,699,591). Approximately 1.7 million individuals were screened, achieving a cumulative coverage of 13.45% in about 6 months. Full-coverage programs could be achieved by AI technology in approximately 1 year, which was 87.5 times more efficient than the manual reading of slides. The sample compliance rate was as high as 99.1%, and compliance rates for positive, negative, and pathology biopsy reviews exceeded 96%. The cost of this program was CN ¥49 (the average exchange rate in 2022 is as follows: US $1=CN ¥6.7261) per person, with the primary screening institution and the third-party testing institute receiving CN ¥19 and ¥27, respectively.

**Conclusions:**

AI-assisted diagnosis has proven to be accessible, efficient, reliable, and low cost, which could support the implementation of full-coverage screening programs, especially in areas with insufficient health resources. AI technology served as a crucial tool for rapidly and effectively increasing screening coverage, which would accelerate the achievement of the World Health Organization’s goals of eliminating cervical cancer.

## Introduction

Cervical cancer can be largely prevented through early intervention [1]. However, it remains the fourth leading cause of cancer-related deaths in women worldwide. The Global Cancer Statistics for 2020 reported approximately 600,000 new cases of and 340,000 deaths from cervical cancer [2]. In response to this significant health challenge, the World Health Organization (WHO) launched the Global Strategy for the Elimination of Cervical Cancer in 2020, highlighting 3 key interventions: human papillomavirus (HPV) vaccination, cervical cancer screening, and cervical disease treatment [3]. The target for coverage of cervical cancer screening in specific populations is to reach 70% and above. In 2021, WHO updated its cervical cancer screening guidelines, providing recommendations to different countries and regions [4]. However, many countries with the highest burden of disease face limitations in achieving sufficient screening coverage [5]. In low- and middle-income countries (LMICs), only 9%-11% of women have been screened, in contrast to the 84% screening rate in high-income countries [6].

The national cervical cancer screening program implemented by the Chinese government in 2009 has played a crucial role in cervical cancer prevention and control (Multimedia Appendix 1). However, there still exists a considerable gap between the current cervical cancer screening rate and the target set by the WHO [7,8]. The economic burden of cervical cancer disease accounts for 18.2% of the global burden [2], and its incidence and mortality rates are 11.4% and 3.4%, respectively [9], with both being the highest in the central region, including Hubei province. The screening rate for Chinese women aged 35-64 years is approximately 36.8%, with lower rates observed in rural areas compared to urban areas [10]. Affordability and service accessibility are still the key factors influencing Chinese women’s use of cervical cancer screening services, followed by education, health insurance, occupation, and family income [11]. Organized free screening can significantly increase their willingness to be screened. While China has already screened 130 million individuals, there are still 300 million people to be screened. Screening technology is a critical factor affecting the increase of screening coverage. Currently, cytology screening is the primary method used in China’s national screening program. However, the shortage of pathologists in primary health institutions has limited the widespread adoption of cytology screening [12]. Studies have also indicated higher cervical cancer mortality rates in rural areas compared to urban areas [9,13]. Therefore, the selection of appropriate technology to rapidly enhance screening coverage, particularly in regions with limited health resources, is an urgent issue in China today.

Artificial intelligence (AI) technology has the potential to address the issue of insufficient pathologists in primary health institutions and accelerate the improvement of screening coverage rates. Therefore, this technology has been implemented in the Hubei government’s organizational screening for 5 years. Now, Hubei province has become the first in China to implement full-coverage of cervical cancer screening for women of appropriate age by adopting AI technology, namely completing cervical cancer screening for 12.67 million urban and rural women in 3 years [14]. AI technology has a wide range of applications in cancer screening with favorable diagnostic performance [15-19]. A few studies conducted on Chinese populations have explored the use of AI technology in cervical cancer screening and diagnosis [11,20,21], comparing its compliance to manual reading, identifying high-grade lesions, and developing AI algorithms [22-24]. Further, 1 recent study compared AI technology with traditional cytology screening and HPV testing, demonstrating comparable cost-effectiveness of AI technology versus HPV testing [25]. Another study focused on the application of AI technology in population screening, including the roles and costs of health care workers, as well as measures to overcome challenges in online screening [26]. Although previous studies have reported results of internal quality control, such as compliance rates between AI and manual groups, evidence of external quality control is lacking. Furthermore, the screening efficiency of AI for ultralarge populations, the speed of improving coverage, the quality of screening, and the responsibilities and costs of the different screening organizations involved are not known. The performance of HPV testing for screening in the population has been reported [27-29], but the performance of AI technology in real-world screening is lacking and there is an urgent need to understand the performance of AI technology in full-coverage screening.

We described the governing and implementing agencies of the full-coverage screening program and their responsibilities, as well as how AI technology made the program a reality. More importantly, this study aimed to evaluate the performance of an AI and cloud diagnostic system in the first full-coverage cervical cancer screening program in China. The evaluation encompassed 4 key dimensions: accessibility of screening services, screening efficiency, diagnostic quality, and cost of different institutions. The assessment provided real-world evidence of the adoption of AI technology to improve screening coverage for other provinces in China and LMICs.

## Methods

### Full-Coverage Screening Program

**Figure 1:**
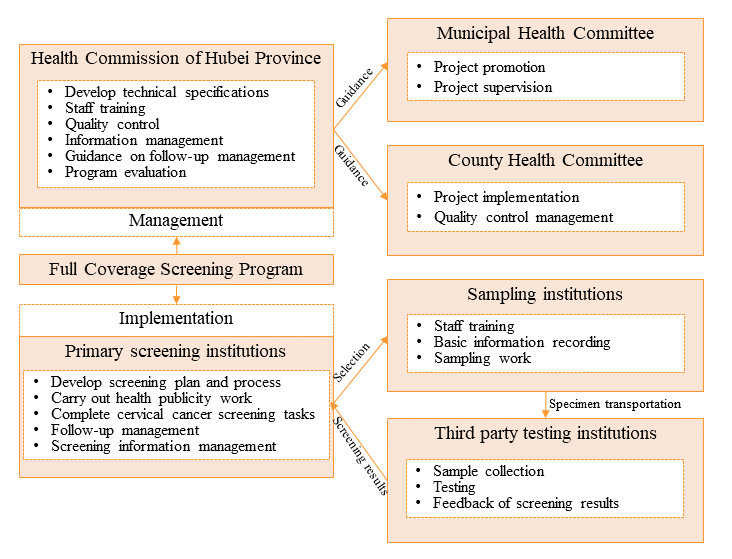
Management and implementation of the full-coverage program.

The full-coverage screening program consists of 2 interconnected systems, a management system led by the provincial health committee and an implementation system led by the maternal and child health hospitals at the county and district levels (Figure 1). These 2 systems work collaboratively to ensure the successful implementation and coordination of the screening program. The health commission is responsible for program development, implementation guidance, project quality control, and evaluation. The cervical cancer screening implementation body consists of primary screening institutions, sampling institutions, and third-party testing institutions. In the implementation of the screening program, the key technology that makes the full-coverage plan a reality is the application of the AI and cloud diagnostic platform.

### AI and Cloud Diagnostic System

#### Principles of AI-Assisted Liquid-Based Cytology

The DNA content of cervical epithelial exfoliated cells is correlated with cervical carcinogenesis. In the AI-assisted diagnostic process, images of cell samples are captured, and the cell nuclei within these images are segmented. For accurate diagnosis, normal cells’ nuclei within a single sample group are the reference in the AI’s algorithm. The segmented nuclei are then quantified based on their cellular DNA content, which ranges from 0 to 255 pixels. Then, by analyzing the pixels, the AI obtains the information on the cell nucleus DNA, and finally, the parameters such as the DNA content of the cell nucleus in this sample are counted to figure out the type of cell lesion. The specific algorithms involved in AI diagnosis are reported in previous studies [24].

#### Screening Procedure

##### Step 1: Sample Acquisition

**Figure 2:**
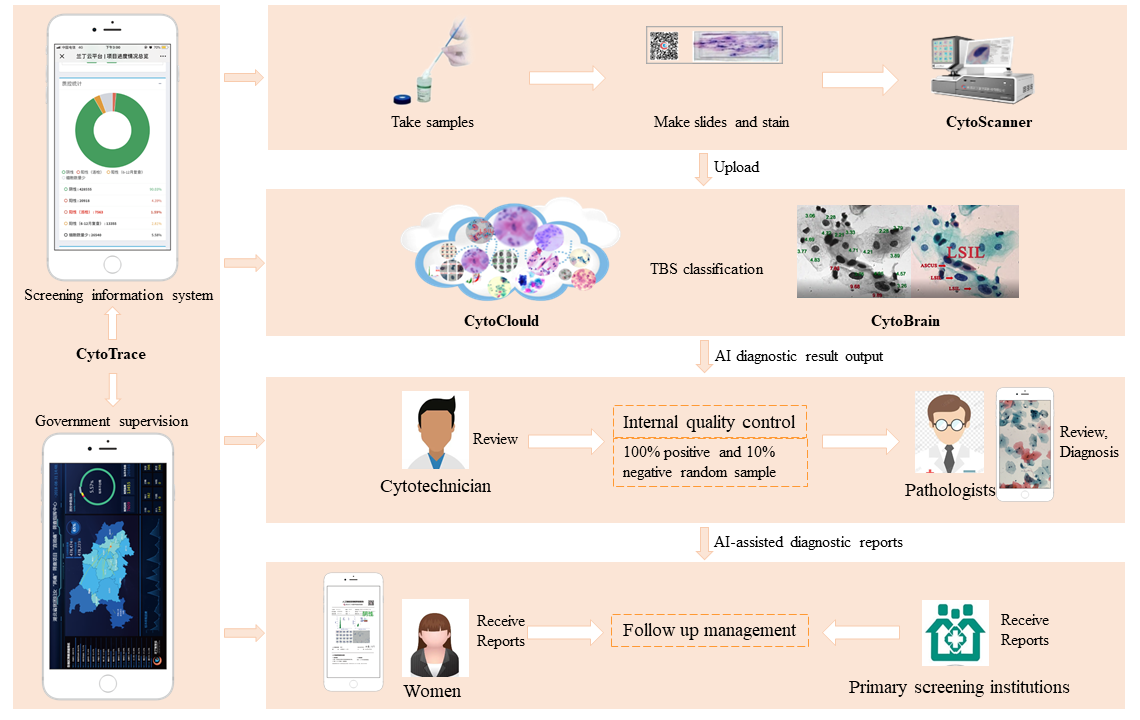
Screening process and quality control. TBS: The Bethesda System.

Women can access free cervical cancer screening services at primary health institutions by presenting their ID cards (Figure 2). At the same time, they are required to sign an informed consent form. Labels with QR codes are automatically produced through identification of the ID card. The staff of the primary health institutions uses a special cervical brush to collect cervical exfoliated cells, which are preserved in sample preservation liquids. Cell samples are transported to the Landing laboratory, a government-appointed testing facility. The information of the whole process from sample collection to diagnostic report is available through a QR code.

##### Step 2: AI-Assisted Liquid-Based Cytology Diagnosis

Laboratory technicians make slides of the samples. The slides are scanned and the cell images are uploaded to the cloud platform. Trained and validated algorithms within the AI diagnostic engine analyze the cellular features present in the images. The results of the AI diagnosis are reviewed by cytotechnicians who identify negative cases and retain suspicious or positive cases for further evaluation.

##### Step 3: Quality Control

To ensure accuracy, all suspicious or positive cases, as well as a randomly selected 10% of negative cases, are mixed. The pathologist then diagnoses these cases and diagnostic reports will be issued. The entire process from AI reading to cytotechnicians review and pathologist review belongs to the internal quality control of our AI-assisted diagnostic system. Importantly, pathologists have the flexibility to work from any location, including their homes or even abroad, as long as they can connect to the cloud platform using their electronic devices.

##### Step 4: Results Notification

Diagnostic reports with negative screening results are sent to the primary health institutions in a centralized file bag. For positive screening results, the diagnostic reports are sent to the primary health institutions in a separate packet. Facility staff are responsible for notifying examinees. They can also obtain the information by scanning the receipt with a QR code through WeChat, which is the fastest way.

##### Step 5: Follow-Up Management

Based on the results of cytological screening feedback, primary health institutions follow up on examinees with abnormal initial screening results. Those with normal cytology results will be screened once every 3 years. If the cytology results are abnormal or suspicious, colposcopy is performed directly. If the colposcopy is negative, then a cytology reexamination is needed after 1 year. If colposcopy is positive, a histopathologic biopsy is performed and follow-up treatment is provided for those patients who require therapy.

#### Quality Control Process

Quality control consists of internal quality control and external quality control (Figure 2). Internal quality control (for details see step 3) is completed by 4 pathologists with senior titles from the landing laboratory, and the frequency of rechecking is daily. Besides, the internal diagnostic quality control is performed using a fully digital process, involving quality control in making slides, staining, and diagnosis. The external quality control is to invite pathologists from tertiary care hospitals to randomly select slides for a recheck regularly, including to recheck the results of cytology diagnosis and pathology diagnosis. That is, external quality control is a reexamination of internal quality control. Quality control adheres to the “Quality Assurance and Quality Control Guidelines for Cervical Cancer Screening” and the “Cervical Cancer Screening Quality Assessment Manual (2022 Edition).”

#### Implementation Stage

The application of the AI and cloud diagnostic system has progressed through 3 stages.

##### Phase 1: Pilot Phase 

This phase was mainly used in some municipal hospitals to improve the efficiency of institutional diagnosis and save the expenditure on institutional labor costs.

##### Phase 2: Provincial Organizational Screening Phase

In 2017, it was first applied in the cervical screening program organized by the provincial government, with a cumulative screening quantity of 1.46 million completed in the first 3 years.

##### Phase 3: Full-Coverage Program Phase

AI-assisted diagnostics was applied in the provincial government’s full-coverage screening program. The technology was also being expanded in some countries in the Belt and Road Initiative, such as Pakistan.

### Data Collection

We collected data on screening quantity, screening time, diagnostic quality, and cost. The screening data were collected from the initiation of the full-coverage screening program until January 31, 2023, with a total of 1,704,461 individuals and 18,261 histopathology biopsies. The screening program followed a closed-loop management process, encompassing screening, follow-up, and treatment for all examinees. We clarified the time and workload of AI-assisted diagnosis through the Wuhan landing cloud intelligence laboratory. Additionally, the daily workload of pathologists was estimated based on guidelines and literature studies, such as the Guidelines for Construction and Management of Pathology Departments (Trial) and literature studies. To evaluate the diagnostic quality, an external quality assessment was organized. The sample size was 220, including 170 randomly selected cytology samples and 50 histopathology samples. Data on the cost of full-coverage screening were obtained from experts of the Health Commission of Hubei Province.

### Ethical Considerations

This study did not expose women’s privacy or identifying information, nor did it result in associated economic or social risks. Women participated in cervical cancer screening voluntarily and signed an informed consent form before being screened. The consent included that their screening information could be used for big data research. This study was conducted in compliance with the Declaration of Helsinki principles and approved by the ethics committee of Tongji Medical College, Huazhong University of Science and Technology (number [2023]IEC(A235)).

## Results

### Accessibility

Cervical cancer screening services were extended to all administrative districts, including 13 prefecture-level administrative regions and 4 directly administered county-level administrative regions in Hubei Province, and a total of 1,704,461 women had been screened (Table 1). The number of county administrative districts covered by the screening program has increased from 83 in 2020 to 103 at present, of which 39 are in urban areas and 64 are in rural areas. The completed screening coverage rate was 13.45% (Multimedia Appendix 2). Among them, Huanggang city had the highest coverage rate of 18.91%.

**Table 1:** Accessibility of cervical cancer screening services in Hubei province.

Initiated cities or counties	Women screened, n	Women aged 35-64 years, n	Screening coverage, %
Wuhan	223,603	2,731,324	8.19
Huangshi	71,423	541,774	13.18
Shiyan	65,536	704,131	9.31
Yichang	124,646	854,964	14.58
Xiangyang	171,260	1,154,377	14.84
Ezhou	28,879	236,836	12.19
Jingmen	81,348	569,827	14.28
Xiaogan	161,670	937,020	17.25
Jingzhou	185,026	1,147,845	16.12
Huanggang	244,097	1,290,808	18.91
Xianning	95,935	583,297	16.45
Enshi	128,674	758,358	16.97
Suizhou	61,633	449,363	13.72
Xiantao	35,249	248,983	14.16
Tianmen	15,986	254,233	6.29
Qianjiang	6986	194,529	3.59
Shennongjia	2510	14,607	17.18
Total	1,704,461	12,672,278	13.45

The screening project was officially launched in September 2022, with 1.54 million individuals screened in 5 months. Before the launch of the project, there were screening pilots in some areas in July (n=155,103) and August (n=9417). The amount of screening was limited, except for Wuhan, where a total of 153,747 individuals were screened in July. A screening peak was rapidly formed after the launch, including a screening volume of about 498,909 in November. The COVID-19 outbreak affected accessibility to screening services, most significantly in December (n=295,199) and January (n=87,760).

The highest active participation after the launch of this project was among rural women with 67.54% (n=1,147,839) of the total screening population. This was followed by urban nonunion women with 27.91% (n=474,420; Table 2). In total, 160,376 (94.07%) of these screenings were normal. Further, 199,473 (5.85%) were cytologically positive, indicating a need for referral for colposcopy. The positive rate of AI-assisted cytological screening was 5.84%. In the pathological examination performed in the project, 1863 individuals were examined for precancerous lesions and 93 individuals were examined for cervical cancer.

**Table 2:** Basic characteristics of examinees.

Characteristics			Examinees, n (%)
**Population categories^a^**			
	Urban nonunion	474,420 (27.91)	
	Urban unions	65,184 (3.84)	
	Rural women of appropriate age	1,147,839 (67.54)	
	Women workers in difficulty	12,148 (0.71)	
**Cytology screening results**			
	NILM^b^	1,603,376 (94.07)	
	ASC-US^c^	68,637 (4.03)	
	LSIL^d^	17,977 (1.05)	
	ASC-H^e^	9613 (0.56)	
	HSIL^f^	2129 (0.12)	
	AGC^g^	1117 (0.07)	
	Failure	1612 (0.09)	
**Histopathological results^h^**			
	Cervicitis	10,645 (58.29)	
	CIN1^i^	5660 (31)	
	CIN2/3^j^	1863 (10.2)	
	CA^k^	93 (0.51)	

^a^At the beginning of project implementation, 4870 women were not classified in the population categories.

^b^NILM: negative for intraepithelial lesion or malignancy.

^c^ASC-US: atypical squamous cells of undetermined significance.

^d^LSIL: low-grade squamous intraepithelial lesion.

^e^ASC-H: atypical squamous cells, cannot exclude a high-grade lesion.

^f^HSIL: high-grade squamous intraepithelial lesion.

^g^AGC: atypical glandular cell.

^h^Some biopsy results were not available because women were screened at program sites.

^i^CIN1: cervical intraepithelial neoplasia grade 1.

^j^CIN2/3: cervical intraepithelial neoplasia grade 2 and 3, including cervical cancer in situ.

^k^CA: cervical cancer.

### Screening Efficiency

Based on real-world screening volumes, full-coverage programs could be achieved in approximately 1 year by using AI technology (Table 3). For the number of pathologists involved in full coverage, AI technology was 87.5 (35,000/400) times more efficient than manual reading of slides. Based on the screening capacity of primary health institutions [12], it would take approximately 16 years to complete full coverage of the program’s screening population. This would be even longer if repeat screening every 3 years is taken into account.

**Table 3:** AI^a^-assisted LBC^b^ versus manual cytology diagnosis.

Screening Method	Scenes	Screening cases/d	Completed 1.7 million/d^c^	Full coverage of Hubei/d^d^
AI-assisted diagnosis	Real-world	35,000^e^	49	362
Pathologists	Hypothetical scenario^f^	400	4250	31,675
Pathologists	Hypothetical scenario^g^	2100	810	6033
Pathologists	Hypothetical scenario^h^	2200	773	5759

^a^AI: artificial intelligence.

^b^LBC: liquid-based cytology.

^c^Screening volume completed as of January 31, 2023, for the project, approximately 1.7 million.

^d^Cervical cancer full-coverage screening program in Hubei province is about 12.67 million.

^e^The current maximum daily screening volume in the real-world is 35,000 individuals, which could continue to expand.

^f^Workload of each pathologist is 100 individuals per day according to the recommended standards. This artificial intelligence–assisted diagnosis system has 4 pathologists. It is assumed that these 4 pathologists used manual reading to do cytology diagnosis.

^g^The survey of screening capacity in 14 provinces in the national cervical cancer screening program found that it took 1003.64 days for urban areas to test 2,107,648 individuals with cytology screening, which means that the urban screening quantity was about 2100 individuals per day.

^h^The same study as above found that the quantity of cytology screening used in rural areas was 2200 (1,120,136/509.15) individuals per day.

### Diagnostic Quality

A total of 220 individuals were reassessed for quality, including 170 cytology slides and 50 histopathology slides (Multimedia Appendix 3). According to the criteria in the cervical cancer quality assessment handbook [30], the specimen satisfaction rate, the compliance rate of negative and positive smear rechecks, and the recheck rate of pathological examination results all met the quality requirements (Table 4). The AI-assisted diagnosis had a 99% compliance rate for positive smears and a 100% compliance rate for negative smears when compared to the cytologist’s diagnosis. All negative cases and CIN2+ (cervical intraepithelial neoplasia) cases were exact matches, with a small number of deviations mainly in low-grade lesions. Compared to pathologists, AI diagnostic systems were less likely to miss or underestimate the severity of cervical lesions.

**Table 4:** Quality assessment indicators and results.

Evaluation indicators	Rate (%)	Related evaluation criteria (%)
Satisfactory rate of specimens^a^	99.1	≥95
Positive smear recheck compliance rate^b^	99	≥85
Negative smear recheck rate	100	≥95
Compliance rate of pathological examination	96	≥95

^a^The assessment method is on-site assessment.

^b^The judgment criteria for compliance is a difference of 2 levels or less.

### Cost

The per capita cost of screening in the full-coverage program is CN ¥49 (US $6.89; Table 5), while the price of health services in primary health institutions is about CN ¥160 (US $22.48). The primary health institutions and third-party testing institutions would receive CN ¥19 (US $2.67) and CN ¥27 (US $3.79) per case, respectively. The primary health institutions are responsible for only cytology sampling, colposcopy examination, and histopathology sampling throughout the screening process. Ultimately the primary health institutions can obtain the cost of services 2, 6, and 7 in Table 5. Third-party testing laboratories provide consumables and bear the costs of sample transportation, subsequent diagnostics, management of information systems, etc. The staff of the Women’s Union were involved in publicity and advocacy, so they received CN ¥3 (US $0.42) per case.

**Table 5:** Screening program costs.

Number	Service	Cost (CN ¥)^a^
1	Training cost for sampling workers, marketing publicity, and organizational management cost	1.5
2	Sampling cost for primary screening institutions	11
3	Logistics cost for samples	1
4	Consumables cost	5
5	Slides, staining, and examination cost	15
6	Colposcopy (for those with a positive primary cytology screening)	5
7	Histopathological diagnosis cost (for those with positive colposcopy)	3
8	Development operation and maintenance costs for the screening information management system	4
9	External quality control and evaluation cost	0.5
10	Cost for the organization and promotion of screening participants	3
11	Total	49

^a^The average exchange rate in 2022 is as follows: US $1=CN ¥6.7261.

## Discussion

### Principal Findings

The diagnostic system based on an AI and cloud platform can achieve coverage of the whole population for cervical cancer screening, especially in areas with insufficient health resources. Cervical cancer screening services were extended to all administrative districts, the number of which has increased from 83 in 2020 to 103 at present. With the voluntary participation of women, a total of 170,461 individuals were screened between July 2022 and January 31, 2023, achieving a coverage rate of 13.45%. Rural women accounted for the largest proportion of screenings at 67.54% (1,147,839/1,699,591). In the screening program, 1,603,376 (94.07%) were diagnosed as normal, 1612 (1612/1,704,461, 0.09%) samples failed, with 1863 (1863/18,261, 10.20%) cases of precancerous lesions and 93 (93/18,261, 0.51%) cases of cervical cancer. The positive rate of AI-assisted cytological screening was 5.84%. Based on the maximum screening quantity of AI-assisted LBC in the real-world, full-coverage programs could be achieved in approximately 1 year, which was 87.5 times more efficient than manual reading of slides. Compared to real-world screening capacity, AI technology could accomplish full-coverage screening amounts at least 15 years ahead of schedule. The review compliance rates of positive and negative smears and pathological biopsies were 96% and above, meeting national quality control requirements. From a public payment perspective, the cost of this program was CN ¥49 (US $6.89) per person, with the primary screening institution and the third-party testing institute receiving CN ¥19 (US $2.67) and CN ¥27 (US $3.79), respectively. In short, AI-assisted diagnosis has proven to be accessible, efficient, reliable, and cost-effective, and plays an important role in facilitating improvements in screening coverage, especially in areas with insufficient health resources. To our knowledge, this study reports the largest sample of AI-assisted cervical cancer screening and is the first to evaluate the performance of AI technology in a full cervical cancer screening program. AI technology plays an important role in promoting screening coverage and opening up new pathways for the elimination of cervical cancer in China and other LMICs.

AI technology has greatly enhanced the accessibility of screening services, especially for rural women. Improving the equity and accessibility of screening services is an important issue for the elimination of cervical cancer [31]. China mainly adopts cytology screening, and the lack of pathologists limits the large-scale application of this technique, especially in remote or rural areas. The full-coverage screening covered all county-level administrative regions in China, with the largest number of rural women screened at 1,147,839 (67.54%), far more than urban women. This is on the one hand because screening services do increase the accessibility of screening services for rural women, and on the other hand, because of the long-term implementation of the screening program in rural areas, rural women have a high level of recognition and willingness to participate in the program. Previous studies have also shown that tissue-based screening can increase participation in screening [32,33]. In addition, according to the geographical characteristics of the county, the implementers adopted the approach of going to the village for screening or organizing women to be screened at the township health center for remote areas to ensure the convenience and accessibility of screening services. AI technology can not only solve the screening problem in remote or poor areas of China but also solve the cervical cancer screening problem in Pakistan by establishing laboratories and exporting equipment abroad. This technology will benefit more Belt and Road Initiative countries in the future.

AI-assisted LBC has greatly enhanced screening efficiency and accelerated the achievement of WHO’s screening goals. With a real-world maximum daily diagnostic workload of 35,000 cases, the AI system will achieve full cervical cancer screening coverage in less than a year, 2 years earlier than expected. Further, the daily diagnostic volume can be continuously increased, which can be achieved by adding high-throughput scanners and technical staff. This screening was completed in about 6 months with a coverage of 13.45% (1,704,461/12,672,278) of women in Hubei, which was huge progress. A survey of the screening capacity of primary health institutions found that some women may not be able to access screening services once in their lifetime [12]. In contrast, AI screening efficiency in the real world was approximately 16 times more efficient than the screening efficiency of primary health institutions. The coverage of screening has a significant impact on cervical cancer incidence and mortality [34-36], so the high efficiency of AI technology also accelerates the process of achieving the goal of cervical cancer elimination.

Diagnostic quality is an important evaluation indicator, including the consistency between AI and manual reading, the failure rate, and the positive cytology screening rate. The national cervical cancer cytology screening positivity rate is 4%, while HPV testing is as high as 10.1% [28]. The cytology screening misdiagnosis rate was lower than that of HPV testing and the leakage rate was higher than that of HPV testing. The positive rate of AI-assisted cytological screening was 5.84%, which was higher than traditional manual reading. This may be attributed to the improved diagnostic performance of AI-assisted cytological screening.

The total number of samples screened this time was about 1.7 million, with only 0.9% (1612/1,704,461) failure samples. Compared to the 3.3% [23] and 2.91% [26] in the previous study, the full-coverage program demonstrated better sample pass rates with the largest sample sizes. This may be due to the continuous training of technicians and strict quality control during the implementation of the full-coverage program. In a previous study with a sample of about 0.7 million, the overall agreement rate between AI and manual reading was 94.7% [23], which belonged to internal quality control. In the census population, AI was significantly more sensitive than manual reading for CIN2+/CIN3+ respectively, while the corresponding specificity was slightly reduced when compared to cytotechnicians. In suspicious populations, AI-assisted reading has similar sensitivity in detecting CIN2+/CIN3+, but is more specific than manual reading [22]. Our study adds another aspect regarding the rate of agreement, namely the consistency of external quality assessments. External quality control was a reevaluation of the outcomes of internal quality control. Additionally, the results of the external quality evaluation showed that the compliance rates for cytology screening and histopathology were 99%-100% and 96%, respectively. This all met the requirements for quality control of cervical screening in China and the WHO.

AI-assisted LBC has a favorable price advantage to support large-scale screening. Price is a very important consideration for the government to purchase this screening service and provide it free to women, especially for the population group. The per capita cost of this full-coverage screening program is CN ¥49 (US $6.89). In comparison, hospitals may charge almost 3 to 4 times (about CN ¥160 [US $22.48]) as much. CN ¥3 (US $0.42) of the project cost is for advocacy and mobilization. However, the cost of the project will continue to decrease over time with the widespread use of social media and the increased willingness to participate. Previous studies have also confirmed that AI-assisted diagnosis is more economical than HPV testing [25]. The affordability of screening services is an important factor affecting the availability of screening services [8,37]. Low cost offers the possibility of achieving screening in large-scale populations, especially in poor areas.

### Policy Implication

The full-coverage screening program in Hubei province is the first provincial coverage program and could provide useful lessons for other cities and regions in China, as well as other LMICs, to rapidly increase screening coverage. First, AI-assisted diagnosis technology has a lower cost and reliable quality to reach as many people as possible with limited government funding and to enhance screening coverage. Second, AI technology can address the challenges of primary health institutions, especially the accessibility of screening services in remote or poor areas. Thus, coverage of vulnerable populations will be achieved and equity in screening services will be improved. Finally, AI technology has tremendous capacity advantages that can rapidly improve screening coverage and shorten the process of achieving the target of 70% screening coverage.

Unlike mass population-based screening, precision screening can be implemented in remote, poor, and financially resource-limited areas, even in LMICs. The target population for precision screening may come from areas with high incidence or mortality of cervical cancer, remote and inaccessible areas, or areas with low screening coverage. The cytology screening approach of AI-assisted diagnosis not only addresses screening in areas with insufficient health resources, but more importantly, through the screening program, improves the screening capacity of staff in the area, follow-up management capacity, and health service providers’ treatment capacity, forming a virtuous cycle of service provision and capacity enhancement.

### Limitation

Women’s participation in screening is a voluntary selection process. Thus, patients who were diagnosed with a positive screening result did not necessarily choose a county or district health service again when they needed further screening or treatment. This is mainly reflected in the absence of pathological results of screened women in the AI information system. This part of the population often chooses to go to the best municipal hospitals for further screening or treatment. This to some extent wastes health resources and makes it more difficult to manage the follow-up of screened women. The challenge of inducing a rational order for medical treatment is also a problem that China’s health care reform is addressing. However, the AI-assisted diagnosis system has facilitated the follow-up management of women’s screening and the overall improvement of women’s health, thanks to the powerful information management system of the AI-assisted diagnosis system, which can keep track of the implementation of the screening program in the whole region and the information of all the test results of screened women.

### Conclusion

Our AI-assisted diagnostic system can complete cervical cancer screening in a short period with low cost and high quality, and make up for the shortage of pathologists in primary health institutions. It has been proven to perform well in large-scale screening and is an effective pathway to address the lack of health resources, especially in areas with insufficient pathologists. AI diagnostics will help China achieve its goal of eliminating cervical cancer ahead of schedule, and also provide a reference for other LMICs and WHO screening guidelines.

## Appendix

Multimedia Appendix 1 Cervical cancer screening policy in China.

Multimedia Appendix 2 Number of women and screening coverage.

Multimedia Appendix 3 Quality assessment indicators and data.

## Abbreviations

AI: artificial intelligence
CIN: cervical intraepithelial neoplasia
HPV: human papillomavirus
LBC: liquid-based cytology
LMIC: low- and middle-income country
WHO: World Health Organization

## References

[1] Cohen PA, Jhingran A, Oaknin A, Denny L (2019). Cervical cancer. Lancet.

[2] Sung H, Ferlay J, Siegel RL, Laversanne M, Soerjomataram I, Jemal A, Bray F (2021). Global cancer statistics 2020: GLOBOCAN estimates of incidence and mortality worldwide for 36 cancers in 185 countries. CA Cancer J Clin.

[3] (2020). Global strategy to accelerate the elimination of cervical cancer as a public health problem. WHO.

[4] (2021). WHO guideline for screening and treatment of cervical pre-cancer lesions for cervical cancer prevention, second edition.

[5] Lemp JM, De Neve JW, Bussmann H, Chen S, Manne-Goehler J, Theilmann M, Marcus ME, Ebert C, Probst C, Tsabedze-Sibanyoni L, Sturua L, Kibachio JM, Moghaddam SS, Martins JS, Houinato D, Houehanou C, Gurung MS, Gathecha G, Farzadfar F, Dryden-Peterson S, Davies JI, Atun R, Vollmer S, Bärnighausen Till, Geldsetzer P (2020). Lifetime prevalence of cervical cancer screening in 55 low- and middle-income countries. JAMA.

[6] Bruni L, Serrano B, Roura E, Alemany L, Cowan M, Herrero R, Poljak M, Murillo R, Broutet N, Riley LM, de Sanjose S (2022). Cervical cancer screening programmes and age-specific coverage estimates for 202 countries and territories worldwide: a review and synthetic analysis. Lancet Glob Health.

[7] GBD 2019 Universal Health Coverage Collaborators (2020). Measuring universal health coverage based on an index of effective coverage of health services in 204 countries and territories, 1990-2019: a systematic analysis for the global burden of disease study 2019. Lancet.

[8] Zhao F, Qiao Y (2019). Cervical cancer prevention in China: a key to cancer control. Lancet.

[9] Zheng R, Zhang S, Zeng H, Wang S, Sun K, Chen R, Li L, Wei W, He J (2022). Cancer incidence and mortality in China, 2016. J Natl Cancer Cent.

[10] Zhang M, Zhong Y, Wang L, Bao Heling, Huang Zhengjing, Zhao Zhenping, Zhang Xiao, Li Chun, Sun Kelly Liang, Wu Jing, Zheng Xiaoying, Wang Linhong (2022). Cervical cancer screening coverage—China, 2018-2019. China CDC Wkly.

[11] Ji L, Chen M, Yao L (2023). Strategies to eliminate cervical cancer in China. Front Oncol.

[12] Li Y, Ma L, Yang C, Chen Z, Zhao Y, Dang L, Lang J, Qiao Y (2019). A study on service capacity of primary medical and health institutions for cervical cancer screening in urban and rural areas in China. Chin J Cancer Res.

[13] Sun K, Zheng R, Lei L, Zhang S, Zeng H, Wang S, Li L, Chen R, Han B, Peng Ji, Wei W, He J (2022). Trends in incidence rates, mortality rates, and age-period-cohort effects of cervical cancer—China, 2003-2017. China CDC Wkly.

[14] 【Hubei Daily】The province will provide free cervical cancer screening for 12.67 million urban and rural women of the appropriate age—Hubei Provincial Health and Wellness Commission.

[15] Wentzensen N, Lahrmann B, Clarke MA, Kinney W, Tokugawa D, Poitras N, Locke A, Bartels L, Krauthoff A, Walker J, Zuna R, Grewal KK, Goldhoff PE, Kingery JD, Castle PE, Schiffman M, Lorey TS, Grabe N (2021). Accuracy and efficiency of deep-learning-based automation of dual stain cytology in cervical cancer screening. J Natl Cancer Inst.

[16] Hu L, Bell D, Antani S, Xue Z, Yu K, Horning MP, Gachuhi N, Wilson B, Jaiswal MS, Befano B, Long LR, Herrero R, Einstein MH, Burk RD, Demarco M, Gage JC, Rodriguez AC, Wentzensen N, Schiffman M (2019). An observational study of deep learning and automated evaluation of cervical images for cancer screening. J Natl Cancer Inst.

[17] Yu K, Hyun N, Fetterman B, Lorey T, Raine-Bennett TR, Zhang H, Stamps RE, Poitras NE, Wheeler W, Befano B, Gage JC, Castle PE, Wentzensen N, Schiffman M (2018). Automated cervical screening and triage,based on HPV testing and computer-interpreted cytology. J Natl Cancer Inst.

[18] Holmström O, Linder N, Kaingu H, Mbuuko N, Mbete J, Kinyua F, Törnquist S, Muinde M, Krogerus L, Lundin M, Diwan V, Lundin J (2021). Point-of-care digital cytology with artificial intelligence for cervical cancer screening in a resource-limited setting. JAMA Netw Open.

[19] Rubin R (2019). Artificial intelligence for cervical precancer screening. JAMA.

[20] Hou X, Shen G, Zhou L, Li Y, Wang T, Ma X (2022). Artificial intelligence in cervical cancer screening and diagnosis. Front Oncol.

[21] Xue P, Ng MTA, Qiao Y (2020). The challenges of colposcopy for cervical cancer screening in LMICs and solutions by artificial intelligence. BMC Med.

[22] Bao H, Bi H, Zhang X, Zhao Y, Dong Y, Luo X, Zhou D, You Z, Wu Y, Liu Z, Zhang Y, Liu J, Fang L, Wang L (2020). Artificial intelligence-assisted cytology for detection of cervical intraepithelial neoplasia or invasive cancer: a multicenter, clinical-based, observational study. Gynecol Oncol.

[23] Bao H, Sun X, Zhang Y, Pang B, Li H, Zhou L, Wu F, Cao D, Wang J, Turic B, Wang L (2020). The artificial intelligence-assisted cytology diagnostic system in large-scale cervical cancer screening: a population-based cohort study of 0.7 million women. Cancer Med.

[24] Chen H, Liu J, Wen QM, Zuo ZQ, Liu JS, Feng J, Pang BC, Xiao D (2021). CytoBrain: cervical cancer screening system based on deep learning technology. J Comput Sci Technol.

[25] Shen M, Zou Z, Bao H, Fairley C, Canfell K, Ong JJ, Hocking J, Chow EPF, Zhuang G, Wang L, Zhang L (2023). Cost-effectiveness of artificial intelligence-assisted liquid-based cytology testing for cervical cancer screening in China. Lancet Reg Health West Pac.

[26] Zhu X, Yao Q, Dai W, Ji L, Yao Y, Pang B, Turic B, Yao L, Liu Z (2023). Cervical cancer screening aided by artificial intelligence, China. Bull World Health Organ.

[27] Bao H, Ma L, Zhao Y, Song B, Di J, Wang L, Gao Y, Ren W, Wang S, Wu J, Wang HJ (2022). Age-specific effectiveness of primary human papillomavirus screening versus cytology in a cervical cancer screening program: a nationwide cross-sectional study. Cancer Commun (Lond).

[28] Zhao Y, Bao H, Ma L, Song B, Di J, Wang L, Gao Y, Ren W, Wang S, Wang HJ, Wu J (2021). Real-world effectiveness of primary screening with high-risk human papillomavirus testing in the cervical cancer screening programme in China: a nationwide, population-based study. BMC Med.

[29] Zhang J, Zhao Y, Dai Y, Dang L, Ma L, Yang C, Li Y, Kong L, Wei L, Zhang S, Liu J, Xi M, Chen L, Duan X, Xiao Q, Abulizi G, Zhang G, Hong Y, Gao X, Zhou Q, Xie X, Li L, Niyazi M, Zhang Z, Tuo J, Ding Y, Si M, Chen F, Song L, Qiao Y, Lang J (2021). Effectiveness of high-risk human papillomavirus testing for cervical cancer screening in China: a multicenter, open-label, randomized clinical trial. JAMA Oncol.

[30] (2022). Cervical cancer quality assessment handbook.

[31] So WKW, Chan DNS, Law BMH, Rana T, Wong CL (2022). Achieving equitable access to cancer screening services to reduce the cancer burden in the Asia-pacific region:experience from Hong Kong. Lancet Reg Health West Pac.

[32] Bao H, Wang L, Brown M, Zhang M, Hunt K, Di J, Zhao Z, Cong S, Fan J, Fang L, Wang L (2020). A nationally quasi-experimental study to assess the impact of partial organized breast and cervical cancer screening programme on participation and inequalities. BMC Cancer.

[33] Gu C, Chan CWH, Chow KM, Yang S, Luo Y, Cheng H, Wang H (2018). Understanding the cervical screening behaviour of chinese women: the role of health care system and health professions. Appl Nurs Res.

[34] Brisson M, Kim JJ, Canfell K, Drolet M, Gingras G, Burger EA, Martin D, Simms KT, Bénard É, Boily MC, Sy S, Regan C, Keane A, Caruana M, Nguyen DTN, Smith MA, Laprise JF, Jit M, Alary M, Bray F, Fidarova E, Elsheikh F, Bloem PJN, Broutet N, Hutubessy R (2020). Impact of HPV vaccination and cervical screening on cervical cancer elimination: a comparative modelling analysis in 78 low-income and lower-middle-income countries. Lancet.

[35] Canfell K, Kim JJ, Brisson M, Keane A, Simms KT, Caruana M, Burger EA, Martin D, Nguyen DTN, Bénard É, Sy S, Regan C, Drolet M, Gingras G, Laprise J, Torode J, Smith MA, Fidarova E, Trapani D, Bray F, Ilbawi A, Broutet N, Hutubessy R (2020). Mortality impact of achieving WHO cervical cancer elimination targets: a comparative modelling analysis in 78 low-income and lower-middle-income countries. Lancet.

[36] Simms KT, Steinberg J, Caruana M, Smith MA, Lew JB, Soerjomataram I, Castle PE, Bray F, Canfell K (2019). Impact of scaled up human papillomavirus vaccination and cervical screening and the potential for global elimination of cervical cancer in 181 countries, 2020-99: a modelling study. Lancet Oncol.

[37] Zhao S, Huang L, Basu P, Domingo EJ, Supakarapongkul W, Ling WY, Ocviyanti D, Rezhake R, Qiao Y, Tay EH, Zhao F (2022). Cervical cancer burden, status of implementation and challenges of cervical cancer screening in Association of Southeast Asian Nations (ASEAN) countries. Cancer Lett.

