# Supplementary material for: Performance of a Full-Coverage Cervical Cancer Screening Program Using on an Artificial Intelligence– and Cloud-Based Diagnostic System: Observational Study of an Ultralarge Population
Source: J Med Internet Res. 2024 Nov 20;26:e51477. doi: 10.2196/51477 (PMC11618014; doi:10.2196/51477)
Supplement: Multimedia Appendix 1 [file jmir_v26i1e51477_app1.docx]

| **Issue time** | **Policy/Information Source** | **Execution time** | **Project objects** | **Project Area** | **Coverage** |
| --- | --- | --- | --- | --- | --- |
| 2009/6/30 | Notice on the Issuance of the Project Management Program for the Examination of Rural Women for "Two Cancers" | 2009-2011 | Rural women aged 35-59 nationwide | 31 provinces | Screening for 10 million rural women |
| 2012/7/30 | National teleconference on "two cancers" screening program for rural women | 2012-2015 | Rural women aged 35-64 nationwide | NA | Screening for 11 million rural women |
| 2015/7/17 | Notice on the Issuance of the Project Management Program for the Examination of Two Types of Cancer in Rural Women (2015 Version) | NA | Rural women aged 35-64 | 31 provinces | Screening for 12 million rural women |
| 2019/9 | Newly Included in the Specifications of Work Related to Basic Public Health Services (2019 Edition) | NA | Women aged 35-64 | NA | Integrating "two cancers" screening into basic public health service projects |
| 2021/12/31 | Notice of the General Office of the National Health and Health Commission on the issuance of the work program for cervical cancer screening and breast cancer screening | NA | Women aged 35-64^a^ | NA | Over 50% coverage rate |
| 2023/1/5 | Action Plan for Accelerated Elimination of Cervical Cancer (2023-2030)^b^ | 2023-2030 | NA | NA | Coverage exceeding 50% by 2025 and 70% by 2030 |

NA indicates that it cannot be obtained directly from the policy document.

^a^ Among them, priority was given to rural women and low-income women in urban areas.

^b^ The project also includes continued promotion of pilot HPV vaccination for school-age girls by 2030; and a 90% treatment rate for patients with cervical cancer and precancerous lesions.
