# Supplementary material for: Performance of a Full-Coverage Cervical Cancer Screening Program Using on an Artificial Intelligence– and Cloud-Based Diagnostic System: Observational Study of an Ultralarge Population
Source: J Med Internet Res. 2024 Nov 20;26:e51477. doi: 10.2196/51477 (PMC11618014; doi:10.2196/51477)
Supplement: Multimedia Appendix 2 [file jmir_v26i1e51477_app2.docx]

| **Initiated**  **cities/counties** | **Population**^a^ | **Women**^b^ | **Women**  **aged 35-64**^c^ | **Women**  **screened** | **Screening**  **coverage** |
| --- | --- | --- | --- | --- | --- |
| Wuhan | 12447718 | 6047456 | 2731324 | 223603 | 8.19% |
| Huangshi | 2469079 | 1199549 | 541774 | 71423 | 13.18% |
| Shiyan | 3209004 | 1559026 | 704131 | 65536 | 9.31% |
| Yichang | 3896407 | 1892986 | 854964 | 124646 | 14.58% |
| Xiangyang | 5260951 | 2555920 | 1154377 | 171260 | 14.84% |
| Ezhou | 1079353 | 524380 | 236836 | 28879 | 12.19% |
| Jingmen | 2596927 | 1261661 | 569827 | 81348 | 14.28% |
| Xiaogan | 4270371 | 2074668 | 937020 | 161670 | 17.25% |
| Jingzhou | 5231180 | 2541456 | 1147845 | 185026 | 16.12% |
| Huanggang | 5882719 | 2857993 | 1290808 | 244097 | 18.91% |
| Xianning | 2658316 | 1291486 | 583297 | 95935 | 16.45% |
| Enshi | 3456136 | 1679089 | 758358 | 128674 | 16.97% |
| Suizhou | 2047923 | 994939 | 449363 | 61633 | 13.72% |
| Xiantao | 1134715 | 551277 | 248983 | 35249 | 14.16% |
| Tianmen | 1158640 | 562900 | 254233 | 15986 | 6.29% |
| Qianjiang | 886547 | 430710 | 194529 | 6986 | 3.59% |
| Shennongjia | 66571 | 32342 | 14607 | 2510 | 17.18% |
| Total | 57752557 | 28057839 | 12672278 | 1704461 | 13.45% |
| ^a^ The population data is from the seventh census of Hubei Province, which is the latest census data. | | | | | |
| ^b^ The proportion of the female population in the total population is 48.59%. | | | | | |
| ^c^ The proportion of the female population aged 35-64 in the total female population is 45.16%. | | | | | |
