# Supplementary material for: Performance of a Full-Coverage Cervical Cancer Screening Program Using on an Artificial Intelligence– and Cloud-Based Diagnostic System: Observational Study of an Ultralarge Population
Source: J Med Internet Res. 2024 Nov 20;26:e51477. doi: 10.2196/51477 (PMC11618014; doi:10.2196/51477)
Supplement: Multimedia Appendix 3 [file jmir_v26i1e51477_app3.docx]

**Evaluation indicators and their meanings**

| **Evaluation Indicators** | **Indicator Descriptions** | **Related evaluation criteria** |
| --- | --- | --- |
| Satisfactory rate of specimens | Numerator: the number of satisfactory smears in the cytology smears sampled; Denominator: number of cases of cytology smears sampled for evaluation | ≥95% |
| Positive smear recheck compliance rate | Numerator: number of cases where the cytology-positive smear reading results of the evaluated person matched the results of the evaluator Denominator: number of cases of all cytology-positive smears sampled for assessment" | ≥85% |
| Negative smear recheck rate | Numerator: number of cases where the cytology-negative smear reading results of the evaluated person matched the results of the evaluator Denominator: number of cases of all cytology-negative smears sampled for assessment" | ≥95% |
| Compliance rate of pathological examination results | Numerator: number of cases in which the histopathology section readings of the evaluated person matched the results of the evaluator Denominator: number of cases of all sections sampled for evaluation | ≥95% |

**Compliance of AI-assisted diagnosis with cytologist diagnosis**

| **AI-assisted diagnosis** | **Cytology expert diagnosis** | | | | |
| --- | --- | --- | --- | --- | --- |
|  | **NILM** | **ASC-US** | **ASC-H** | **LSIL** | **HSIL** |
| NILM（n=60） | 60 | 0 | 0 | 0 | 0 |
| ASC-H (n=15) | 0 | 0 | 8 | 0 | 7 |
| LSIL (n=40) | 1 | 1 | 1 | 35 | 2 |
| HSIL (n=55) | 0 | 0 | 0 | 1 | 54 |
| Total | 61 | 1 | 9 | 35 | 64 |

**Compliance of pathological diagnosis with external expert quality control**

| **Histopathology** | **Pathologist Quality Control** | | | |
| --- | --- | --- | --- | --- |
|  | **Cervicitis** | **CIN1** | **CIN2/3** | **CA** |
| Cervicitis (n=32) | 32 | 0 | 0 | 0 |
| CIN1 (n=13) | 2 | 11 | 0 | 0 |
| CIN2/3 (n=4) | 0 | 0 | 4 | 0 |
| CA (n=1) | 0 | 0 | 0 | 1 |
| Total | 34 | 11 | 4 | 1 |
